# Increased hepatic mitochondrial FA oxidation reduces plasma and liver TG levels and is associated with regulation of UCPs and APOC-III in rats

**DOI:** 10.1194/jlr.M074849

**Published:** 2017-05-04

**Authors:** Carine Lindquist, Bodil Bjørndal, Christine Renate Rossmann, Deusdedit Tusubira, Asbjørn Svardal, Gro Vatne Røsland, Karl Johan Tronstad, Seth Hallström, Rolf Kristian Berge

**Affiliations:** Departments of Clinical Science* University of Bergen, Bergen, Norway; Departments of Biomedicine,** University of Bergen, Bergen, Norway; Department of Heart Disease,† Haukeland University Hospital, Bergen, Norway; Institute of Physiological Chemistry,§ Medical University of Graz, Graz, Austria

**Keywords:** 2-(tridec-12-yn-1-ylthio)acetic acid, lipids, lipoproteins, metabolic syndrome, mitochondria, nonalcoholic fatty liver disease, fatty acid metabolism, lipids/oxidation, fatty acid, triglyceride, uncoupling protein, apolipoprotein C-III

## Abstract

Hepatic mitochondrial function, APOC-III, and LPL are potential targets for triglyceride (TG)-lowering drugs. After 3 weeks of dietary treatment with the compound 2-(tridec-12-yn-1-ylthio)acetic acid (1-triple TTA), the hepatic mitochondrial FA oxidation increased more than 5-fold in male Wistar rats. Gene expression analysis in liver showed significant downregulation of APOC-III and upregulation of LPL and the VLDL receptor. This led to lower hepatic (53%) and plasma (73%) TG levels. Concomitantly, liver-specific biomarkers related to mitochondrial biogenesis and function (mitochondrial DNA, citrate synthase activity, and cytochrome c and TFAM gene expression) were elevated. Interestingly, 1-triple TTA lowered plasma acetylcarnitine levels, whereas the concentration of β-hydroxybutyrate was increased. The hepatic energy state was reduced in 1-triple TTA-treated rats, as reflected by increased AMP/ATP and decreased ATP/ADP ratios, whereas the energy state remained unchanged in muscle and heart. The 1-triple TTA administration induced gene expression of uncoupling protein (UCP)2 and UCP3 in liver. In conclusion, the 1-triple TTA-mediated clearance of blood TG may result from lowered APOC-III production, increased hepatic LPL gene expression, mitochondrial FA oxidation, and (re)uptake of VLDL facilitating drainage of FAs to the liver for β-oxidation and production of ketone bodies as extrahepatic fuel. The possibility that UCP2 and UCP3 mediate a moderate degree of mitochondrial uncoupling should be considered.

CVD is the leading cause of death worldwide, especially in developed countries (1, 2). Meta-analyses have shown that low HDL-cholesterol and high triglyceride (TG) levels in blood are strongly associated with increased CVD risk (3, 4). Recently, TG-rich lipoproteins were found to causally influence risk of coronary heart disease in individuals with favorable HDL-cholesterol (5). Thus, it is important to consider TG in addition to the traditional risk factors, cholesterol and LDL cholesterol (6–8). TG is transported in chylomicrons (CHYLs) and VLDL from the intestine and liver, respectively, to peripheral tissues where LPL hydrolyzes TG to glycerol and FAs for subsequent uptake. Several lines of evidence have implicated APOC-II and APOC-III in the regulation of CHYL and VLDL metabolism, as LPL uses APOC-II as a cofactor and is inhibited by APOC-III (9). Overexpression of APOC-III in transgenic mice causes hypertriglyceridemia (10) and has also been suggested to be a characteristic feature of hypertriglyceridemia in human patients (11). APOC-III deficiency protects against metabolic disease in mice (12) and it has been proposed that levels of VLDL and APOC-III are better prognostic markers of CVD than plasma TG (13). Accordingly, APOC-III and LPL are among the potential targets for lipid-lowering drugs.

The liver is important for regulating lipid homeostasis and metabolism. CHYL and VLDL remnants are taken up by the liver and, under normolipidemic conditions, TG is either oxidized or secreted as VLDL. However, excessive accumulation of hepatic fat unrelated to alcohol consumption, hepatic steatosis, or nonalcoholic fatty liver disease is the most frequent liver disease in industrialized countries (14). Moreover, the development of this disease often parallels that of insulin resistance and is associated with obesity, metabolic syndrome, dyslipidemia, and type 2 diabetes mellitus (15), and has been linked to mitochondrial malfunction (16). Mitochondria are active in β-oxidation of FAs and, in situations with excess uptake of lipids by the liver, an adaptive increase in mitochondrial oxidative capacity is required. Under these conditions, mitochondrial dysfunction will compromise metabolic performance and may lead to elevated production of reactive oxygen species (17). Hence, mitochondrial adaptations are important to protect against harmful effects of oxidative stress, inflammation, and intrahepatic accumulation of lipids (16, 18).

Given that mitochondrial function and mitochondrial FA oxidation capacity seem to regulate TG levels both in liver and plasma, we have synthesized FA analogs as tetradecylthioacetic acid (TTA), targeting mitochondria (19, 20), which were subsequently shown to interfere with APOC-III gene expression (20). Notably, TTA is blocked for β-oxdation due to the sulfur atom, but can be degraded from the ω-end. Therefore, 2-(tridec-12-yn-1-ylthio)acetic acid (C_15_H_26_O_2_S) (1-triple TTA) is of special interest; in addition to the sulfur atom at the β position, a triple bond is introduced at the ω-1 position. These alterations make the compound resistant to both β-oxidation and ω-oxidation (**Fig. 1**); thus, 1-triple TTA might be a more potent drug than TTA. In this study, we aimed to test how 1-triple TTA influences mitochondrial function and plasma and hepatic TG levels, as well as hepatic *ApoC-III* mRNA levels.

**Fig. 1.**
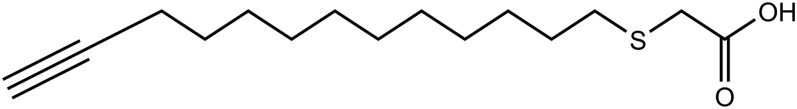
Skeletal structure of 1-triple TTA.

## MATERIALS AND METHODS

### Animal study

The animal study was conducted according to the Guidelines for the Care and Use of Experimental Animals and in accordance with the Norwegian legislation and regulations governing experiments using live animals. The Norwegian State Board of Biological Experiments with Living Animals approved the protocol (permit number 2015-7367). All efforts were made to optimize the animal environment and minimize suffering. Male Wistar rats, 5 weeks old, were purchased from Taconic (Ejby, Denmark). Upon arrival, the rats were randomized using Research Randomizer (21), labeled, and placed in open cages, four in each cage, where they were allowed to acclimatize to their surroundings for 1 week. During the acclimatization and experimental period, the rats had unrestricted access to chow and tap water. The rats were kept in a 12 h light/dark cycle at a constant temperature (22 ± 2°C) and a relative humidity of 55% (±5%). Upon the start of the experiment, the rats were block randomized to their respective interventions (21). During the experiment, there were two rats in each cage separated with a divider that let them have sniffing contact. The control group (n = 8) received 0.5 ml 0.5% methylcellulose (Hospital Pharmacy, Bergen, Norway) daily and the experimental group (n = 5) received 0.02 g 1-triple TTA (Synthetica AS, Oslo, Norway) dissolved in 0.5 ml 0.5% methylcellulose daily. Methylcellulose was given orally by gavage. All animals were weighed daily and feed intake was determined weekly.

At euthanization, rats were anesthetized by inhalation of 5% isoflurane (Schering-Plough, Kent, UK). EDTA-blood was collected by cardiac puncture and immediately placed on ice. The samples were centrifuged and plasma was stored at −80°C prior to analysis. Heart, liver, and epididymal white adipose tissue were collected and weighed. Fresh samples from liver were prepared for β-oxidation analysis (see below). The remaining parts of the liver, heart, and samples from adipose and muscle tissues were immediately snap-frozen in liquid nitrogen and stored at −80°C until further analysis.

### Quantification of plasma and liver lipids

Liver lipids were extracted from frozen samples according to Bligh and Dyer (22), evaporated under nitrogen, and redissolved in isopropanol before analysis. Lipids from liver and plasma were measured enzymatically on a Hitachi 917 system (Roche Diagnostics GmbH, Mannheim, Germany) using the TG kit from Roche Diagnostics (GPO-PAP, 11730711) and NEFA FS kit from Diagnostic Systems GmbH (Holzheim, Germany).

### Quantification of carnitine metabolites and ketone body in plasma

L-carnitine, trimethyllysine, γ-butyrobetaine, and acetylcarnitine were analyzed in plasma by HPLC-MS/MS, as described by Vernez, Wenk, and Krahenbuhl (23) with some modifications (24). The ketone body, β-hydroxybutyrate, was analyzed in plasma by colorimetric assay kit from Cayman Chemical Company (item number 700190; Ann Arbor, MI).

### Measurement of high energy phosphates by HPLC

Freeze-clamped biopsies were taken from the apex of the heart and liver, as well as skeletal muscle, and stored in liquid nitrogen. The sample preparation and HPLC measurement of ATP, ADP, AMP, and phosphocreatine (for heart and muscle samples), as well as hypoxanthine and xanthine, were performed as previously described (25–27). A piece of frozen tissue (50–100 mg) was homogenized with a micro-dismembrator and deproteinized with 500 μl of 0.4 mol/l perchloric acid. After centrifugation (12,000 *g*), 200 μl of the acid extract were neutralized with 20–25 μl of 2 mol/l potassium carbonate (4°C). The supernatant (20 μl injection volume) obtained after centrifugation was used for HPLC analysis. The pellets of the acid extract were dissolved in 1 ml of 0.1 mol/l sodium hydroxide and further diluted 1:10 with physiologic saline for protein determination (BCA protein assay; Pierce). High energy phosphates (ATP, ADP, AMP, and phosphocreatine for heart and muscle samples), hypoxanthine, and xanthine were measured by HPLC, as previously described (25–27). In brief, separation was performed on a Hypersil ODS column (5 μm, 250 × 4 mm internal diameter) using an L-2200 autosampler, two L-2130 HTA pumps, and a L-2450 diode array detector (all VWR Hitachi). Detector signals (absorbance at 214 and 254 nm) were recorded and the program EZchrom Elite (VWR) was used for data requisition and analysis. Energy charge was calculated employing the following formula: energy charge = (ATP + 0.5 ADP)/(AMP + ADP + ATP).

### Mitochondrial FA oxidation and enzyme activities

Post-nuclear fractions from liver, muscle, and heart were prepared (28). Palmitoyl-CoA oxidation was measured in the postnuclear fraction from fresh liver as acid-soluble products, as described by (29). The following enzyme activities were measured in the postnuclear fraction from frozen liver tissue: carnitine *O*-palmitoyltransferase (CPT)-II (EC: 2.3.1.21) (30), mitochondrial HMG-CoA synthase (EC: 2.3.3.10) (31), citrate synthase (EC: 2.3.3.1) (32, 33), FAS (EC: 2.3.1.85) (34), acetyl-CoA carboxylase (EC: 6.4.1.2) (35), malonyl-CoA decarboxylase (EC: 4.1.1.9) (36), and β-ketothiolase (EC: 2.3.1.16) (37). The 3-oxoacid CoA-transferase (EC: 2.8.3.5) activity was measured in frozen heart and muscle tissue (38, 39).

The amount of proteins was measured by Bio-Rad protein assay kit (Bio-Rad Laboratories, Hercules, CA).

### Western blot: nonphosphorylated and phosphorylated AMP-activated protein kinase

Amounts of AMP-activated protein kinase (AMPK) and phosphorylated AMPK (AMPK-P) were analyzed by Western blot. Proteins from frozen liver samples were extracted using a nuclear extract kit (Active Motif, Carlsbad, CA). The amount of protein was determined by Bio-Rad DC™ protein assay reagents. Twenty micrograms of proteins, Laemmli 1× sample buffer (Bio-Rad), and 10 mM DTT were run on Mini-protean TGX Precast gels 4–20% (Bio-Rad), blotted on a PVDF membrane using iBlot gel transfer stack (Invitrogen, Carlsbad, CA), blocked in TBS (pH 8.0) with 3% nonfat milk (catalog number T8793; Sigma-Aldrich), and washed in TBS with Tween20 (Sigma-Aldrich). Blots were incubated with rabbit-anti-AMPK [1:500 or 1:1,000 (catalog number 2532, lot 19)] or rabbit-anti-AMPK-P [1:1,000 (catalog number 2535, lot 14)] and β-actin [1:1,000 (catalog number 4970, lot 7; Cell Signaling Technology, Danvers, MA)], overnight at 4°C, and anti-rabbit IgG HRP-linked secondary Ab [1:1,000 (catalog number 7074, lot 24; Cell Signaling) for 1 h at room temperature and developed with Supersignal™ ELISA Femto substrate for 1 min and analyzed using the FluorChem HD2 program (Alpha Innotech, Kasendorf, Germany). AMPK and AMPK-P were normalized to β-actin.

### Transmission electron microscopy

Frozen liver samples were fixed in cold 0.1 M Na-cacodylate buffer (pH 7.4) containing 2.5% glutaraldehyde 4°C, overnight, and prepared as previously described (40).

### Hepatic gene expression analysis

Tissue samples (20 mg frozen liver) were homogenized in RNeasy lysis buffer from Qiagen (catalog number 79216; Hilden, Germany) with 1% β-mercaptoethanol using Tissuelyser II (Qiagen) for 2 × 2 min at 25 Hz and total cellular RNA was further purified using the RNeasy mini kit (Qiagen), including DNase digestion. cDNA was produced from 500 ng RNA using the high-capacity cDNA reverse transcription kits (Applied Biosystems, Waltham, MA). RT-PCR was performed on Sarstedt 384-well Multiply-PCR plates (Sarstedt Inc., Newton, NC,) using an ABI Prism 7900HT sequence detection system from Applied Biosystems with the software, SDS 2.3. mRNA levels of genes of interest were detected in 1× Taqman buffer by using the following probes and primers listed in alphabetical order: acetyl-CoA carboxylase (*Acaca*) (Rn00573474_m1); medium-chain acyl-CoA dehydrogenase (*Acadm*) (Rn00566390_m1); long-chain acyl-CoA dehydrogenase (*Acadl*) (Rn00563121_m1); very-long-chain acyl-CoA dehydrogenase (*Acadvl*) (Rn00563649_m1); 4-*N*-trimethylaminobutyraldehyde dehydrogenase (*Aldh9a1*) (Rn01491039_m1); *ApoB* (Rn01499054_m1); *ApoC-II* (Rn01764530_g1); *ApoC-III* (Rn00560743); ATP synthase H^+^ transporting mitochondrial F_1_ complex γ polypeptide 1 (*Atp5c1*) (Rn01487287_m1); ATP synthase H^+^ transporting mitochondrial F_0_ complex subunit F2 (*Atp5j2*) (Rn01409509_g1); γ-butyrobetaine hydroxylase (*Bbox1*) (Rn00575255_m1); cluster of differentiation 36 (*Cd36*) (Rn00580728_m1); *Cpt1a* (Rn00580702_m1); *Cpt2* (Rn00563995_m1); carnitine *O*-acetyltransferase (*Crat*) (Rn01758585_m1); somatic cytochrome c (*Cycs*) (Rn00820639_g1); mitochondrial 2,4 dienoyl-CoA reductase 1 (*Decr1*) (Rn00589420_m1); diglyceride acyltransferase (*Dgat*)*1* (Rn00584870_m1); *Dgat2* (Rn01506787_m1); FA binding protein 1 (*Fabp1*) (Rn00664587_m1); *Fasn* (Rn00569117_m1); mitochondrial glycerol-3-phosphate dehydrogenase 2 (*Gpd2*) (Rn00562472_m1); hydroxyacyl-CoA dehydrogenase/3-ketoacyl-CoA thiolase/enoyl-CoA hydratase (trifunctional protein) α subunit (*Hadha*) (Rn00590828_m1); hydroxyacyl-CoA dehydrogenase/3-ketoacyl-CoA thiolase/enoyl-CoA hydratase (trifunctional protein) β subunit (*Hadhb*) (Rn00592435_m1); mitochondrial 3-hydroxy-3-methylglutaryl-CoA synthase 2 (*Hmgcs2*) (Rn00597339_m1); *Lpl* (Rn00561482_m1); microsomal TG transfer protein (large subunit) (*Mttp*) (Rn01522970_m1); NADH dehydrogenase (ubiquinone) 1α subcomplex 9 (*Ndufa9*) (Rn01462923_m1); *Pparα* (Rn00566193_m1); *Pparδ* (Rn00565707_m1); *Pparγ* (Rn00440945_m1); PPARγ coactivator 1α (*Ppargc1a*) (Rn00580241_m1); protein kinase AMP-activated α1 catalytic subunit (*Prkaa1*) (Rn00569558_m1); protein kinase AMP-activated α2 catalytic subunit (*Prkaa2*) (Rn00576935_m1); *Slc25a20* (Rn00588652_m1); solute carrier family 22 (organic cation/carnitine transporter) member 5 (*Slc22a5*) (Rn01471177_m1); mitochondrial transcription factor A (*Tfam*) (Rn00580051_m1); *N*^ε^-trimethyllysine hydroxylase (*Tmlhe*) (Rn00591314_m1); uncoupling protein (*Ucp*)*2* (Rn01754856_m1); *Ucp3* (Rn00565874_m1); VLDL receptor (*Vldlr*) (Rn00565784_m1). A standard curve using either an appropriate cDNA sample or universal rat reference RNA was performed for each probe. The 18S (RT-CKFT-18S) was run for every probe. Data presented are normalized to 18S.

### Mitochondrial DNA quantification

Total DNA was isolated from liver, heart, and muscle tissue using DNAeasy blood and tissue kit and Allprep DNA/RNA Mini kit (both from Qiagen) and analyzed as described previously (41, 42). A specific primer/probe set for mitochondrial DNA (mtDNA), mitochondrially encoded NADH dehydrogenase 1 (Rn03296764_s1), was purchased from Applied Biosystems and nuclear DNA (nDNA) primer 18S (RT-CKFT-18S) from Eurogentec. Quantitative PCR was carried out as above and the mtDNA:nDNA ratio was calculated.

### Statistical analysis

Data were analyzed using Prism software (Graph-Pad Software; San Diego, CA) to determine statistical significance. The results are shown as mean ± SD of 5–8 rats per group. mRNA levels are shown as relative levels compared with control mean with SD, except for *Ucp3*. Student’s *t*-test was used to evaluate statistical differences between the intervention and control. Pearson’s correlation coefficients were used when comparing two independent variables. *P* <0.05 was considered statistically significant.

## RESULTS

### 1-triple TTA decreases free carnitine and acetylcarnitine in plasma

**Fig. 2.**
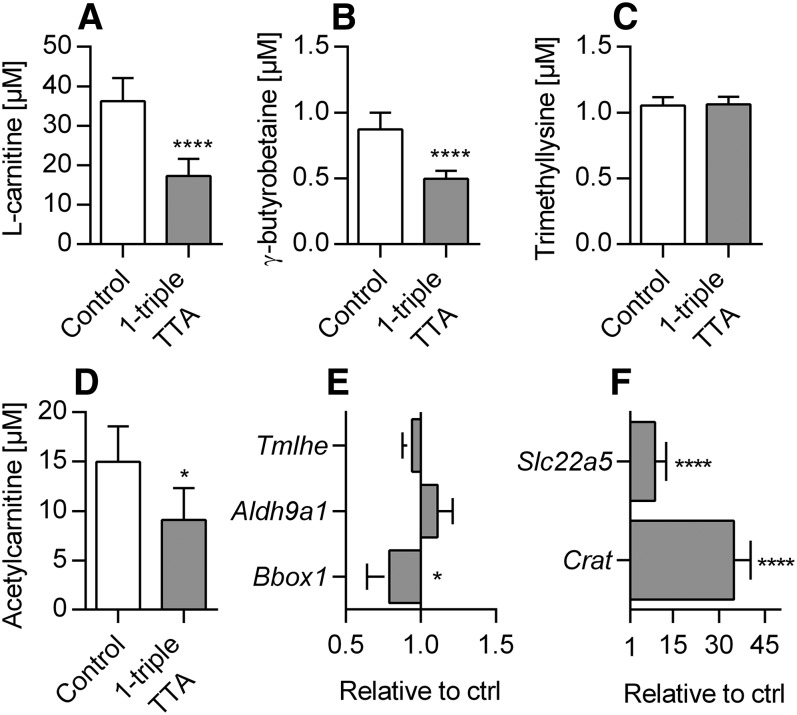
Plasma concentrations of carnitines and hepatic mRNA levels of carnitine metabolism in Wistar male rats given 100 mg/kg 1-triple TTA daily for 3 weeks compared with controls. A: Plasma L-carnitine. B: Plasma γ-butyrobetaine. C: Plasma trimethyllysine. D: Plasma acetylcarnitine. E: Hepatic mRNA levels of enzymes involved in carnitine synthesis, *Tmlhe*, *Aldh9a1*, and *Bbox1*, relative to control. F: Hepatic mRNA levels of the carnitine transporter, *Slc22a5*, and *Crat* relative to control. Values are shown as mean ± SD (n = 5–8). Significant difference compared with controls was determined with Student’s *t*-test (*P ≤ 0.05, ****P ≤ 0.0001).

Carnitine is involved in the generation of metabolic energy from long-chain FAs by mediating their transport across the mitochondrial membrane. The levels of plasma carnitine and its precursor, γ-butyrobetaine, were significantly decreased by 1-triple TTA administration, whereas trimethyllysine remained constant (**Fig. 2**). The short-chain acetylcarnitine level was also decreased (Fig. 2D). The liver is an important site of carnitine production; therefore, we also evaluated whether 1-triple TTA changed the hepatic gene expression of proteins related to carnitine biosynthesis. The mRNA levels of *Tmlhe* and *Aldh9a1* remained constant, whereas *Bbox1* was significantly reduced (Fig. 2E). Interestingly, the mRNA level of *Crat* (a mitochondrial matrix enzyme that catalyzes the interconversion of acetyl-CoA and acetylcarnitine) and the carnitine transporter, *Slc22a5*, were significantly increased by 1-triple TTA compared with controls (Fig. 2F). These results suggest that 1-triple TTA downregulates the levels of carnitine and short-chained acylcarnitines by reduced biosynthesis of carnitine and/or increased consumption.

### 1-triple TTA changes energy state and mitochondrial function in liver

**Fig. 3.**
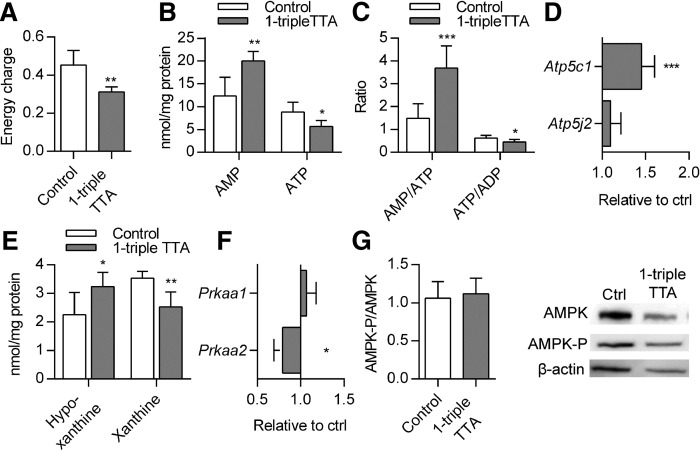
Hepatic energy parameters in Wistar male rats given 100 mg/kg 1-triple TTA for 3 weeks compared with controls. A: Energy charge {[(ATP) + 1/2(ADP)]/[(ATP) + (ADP) + (AMP)]}. B: Adenosine mono- and triphosphates (AMP, ATP). C: Ratio of AMP/ATP and ATP/ADP. D: Hepatic mRNA levels of genes of ATP synthase, *Atp5c1* and *Atp5j2*, relative to control. E: Purine degradation products, hypoxanthine and xanthine. F: Hepatic mRNA levels of AMPK catalytic subunits, *Prkaa1* and *Prkaa2*, are shown relative to control. G: Quantitative results of the ratio between AMPK-P and AMPK related to β-actin in liver from Western blot and representative bonds. Protein levels were quantified by normalizing to one bond on each membrane. Electrophoresis and the Western blot procedure were repeated four times. Values are shown as mean ± SD (n = 5–8 if not otherwise stated). Significant difference compared with controls was determined with Student’s *t*-test (*P ≤ 0.05, **P ≤ 0.01, ***P ≤ 0.001).

**Fig. 4.**
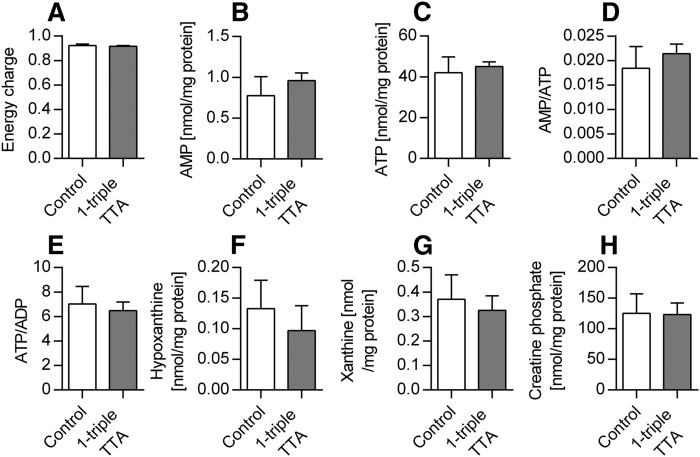
Muscle energy parameters in Wistar male rats given 100 mg/kg 1-triple TTA for 3 weeks compared with controls. A: Energy charge {[(ATP) + 1/2(ADP)]/[(ATP) + (ADP) + (AMP)]}. B: AMP. C: ATP. D: Ratio of AMP/ATP. E: Ratio of ATP/ADP. F: Purine degradation product, hypoxanthine. G: Purine degradation product, xanthine. H: Creatine phosphate. Values are shown as mean ± SD (n = 5–7). Significant difference compared with controls was determined with Student’s *t*-test.

**Fig. 5.**
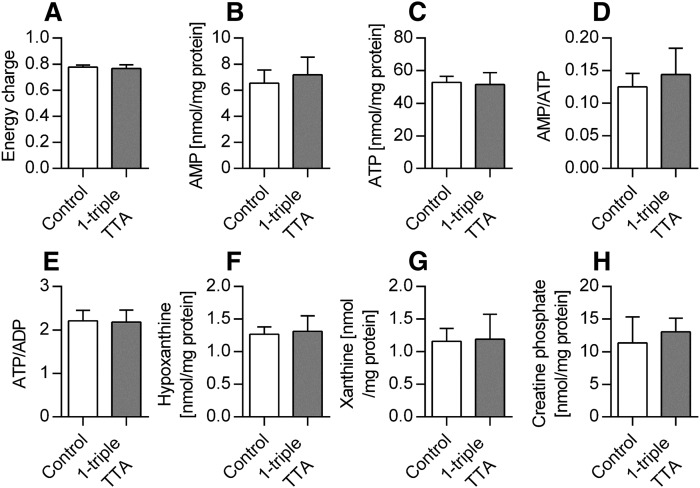
Heart energy parameters in Wistar male rats given 100 mg/kg 1-triple TTA for 3 weeks compared with controls. A: Energy charge {[(ATP) + 1/2(ADP)]/[(ATP) + (ADP) + (AMP)]}. B: AMP. C: ATP. D: Ratio AMP/ATP. E: Ratio ATP/ADP. F: Purine degradation product, hypoxanthine. G: Purine degradation product, xanthine. H: Creatine phosphate. Values are shown as mean ± SD (n = 5–8). Significant difference compared with controls was determined with Student’s *t*-test.

The ability of 1-triple TTA to influence the carnitine and acetylcarnitine plasma levels prompted us to investigate the hepatic energy status, as well as mitochondrial biogenesis and function. The 1-triple TTA treatment significantly decreased energy charge (**Fig. 3A**) and ATP concentrations (Fig. 3B) of the liver compared with controls. The decreased energy charge was also reflected by significantly increased AMP concentrations (Fig. 3B), significantly increased AMP/ATP ratio, and significantly decreased ATP/ADP ratio (Fig. 3C) compared with controls. The hepatic ADP level was not changed by 1-triple-TTA (data not shown). In accordance with the decreased hepatic ATP concentrations, the mRNA level of the F_0_ hepatic ATP synthase subunit, *Atp5c1*, was significantly increased after 1-triple TTA administration, whereas the mRNA level of an F_1_ subunit, *Atp5j2*, remained constant (Fig. 3D). Furthermore, the hepatic content of the adenine degradation products, xanthine and hypoxanthine, was altered. Hypoxanthine levels were significantly increased, whereas xanthine levels were significantly decreased after 1-triple TTA administration (Fig. 3E). It is noteworthy that the hepatic mRNA level of the catalytic subunit of AMPK, *Prkaa1*, was unchanged, whereas the catalytic subunit, *Prkaa2*, of AMPK was significantly decreased after 1-triple TTA treatment (Fig. 3F). No difference in AMPK-P compared with nonphosphorylated AMPK was observed (Fig. 3G). The effect of 1-triple TTA on tissue energy status was found to be specific for liver because no changes were seen in samples from muscle (**Fig. 4**) and heart (**Fig. 5**) . Also, the creatine phosphate levels were unchanged in these two tissues compared with control (Figs. 4H, 5H).

**Fig. 6.**
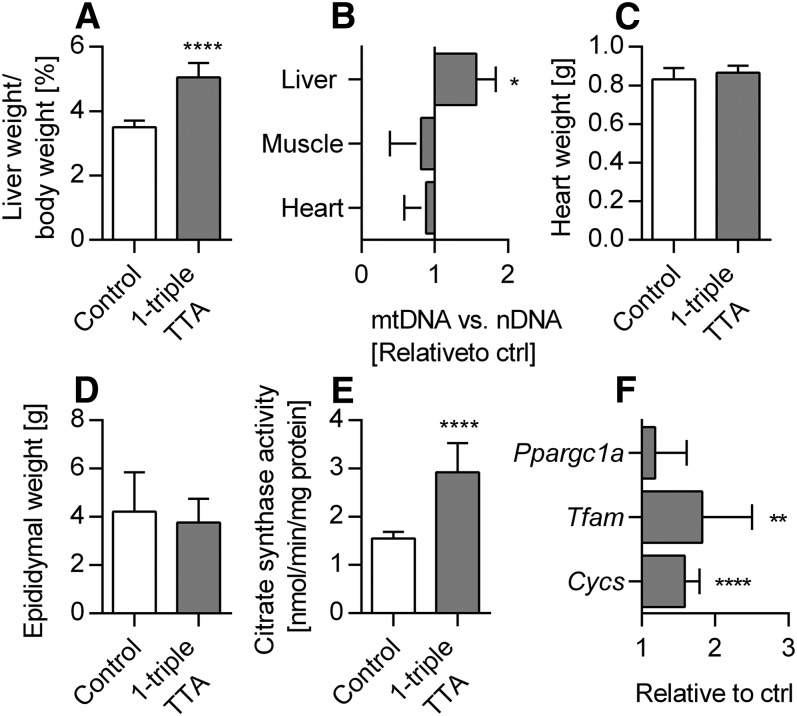
Organ weights and mitochondrial biomarkers in Wistar male rats given 100 mg/kg 1-triple TTA for 3 weeks compared with controls. A: Liver index [(liver weight/body weight) × 100]. B: mtDNA (NADH dehydrogenase 1) compared with nDNA (18S) relative to control. C: Heart weight. D: Epididymal WAT weight. E. Hepatic citrate synthase activity. F. Hepatic mRNA levels of PGC-1α (*Ppargc1a*) mitochondrial transcription factor A (*Tfam*) and cytochrome c (*Cycs*). Values are shown as mean ± SD (n = 4–8). Significant difference compared with controls was determined with Student’s *t*-test (*P ≤ 0.05, **P ≤ 0.0001 ****P ≤ 0.0001).

**Fig. 7.**
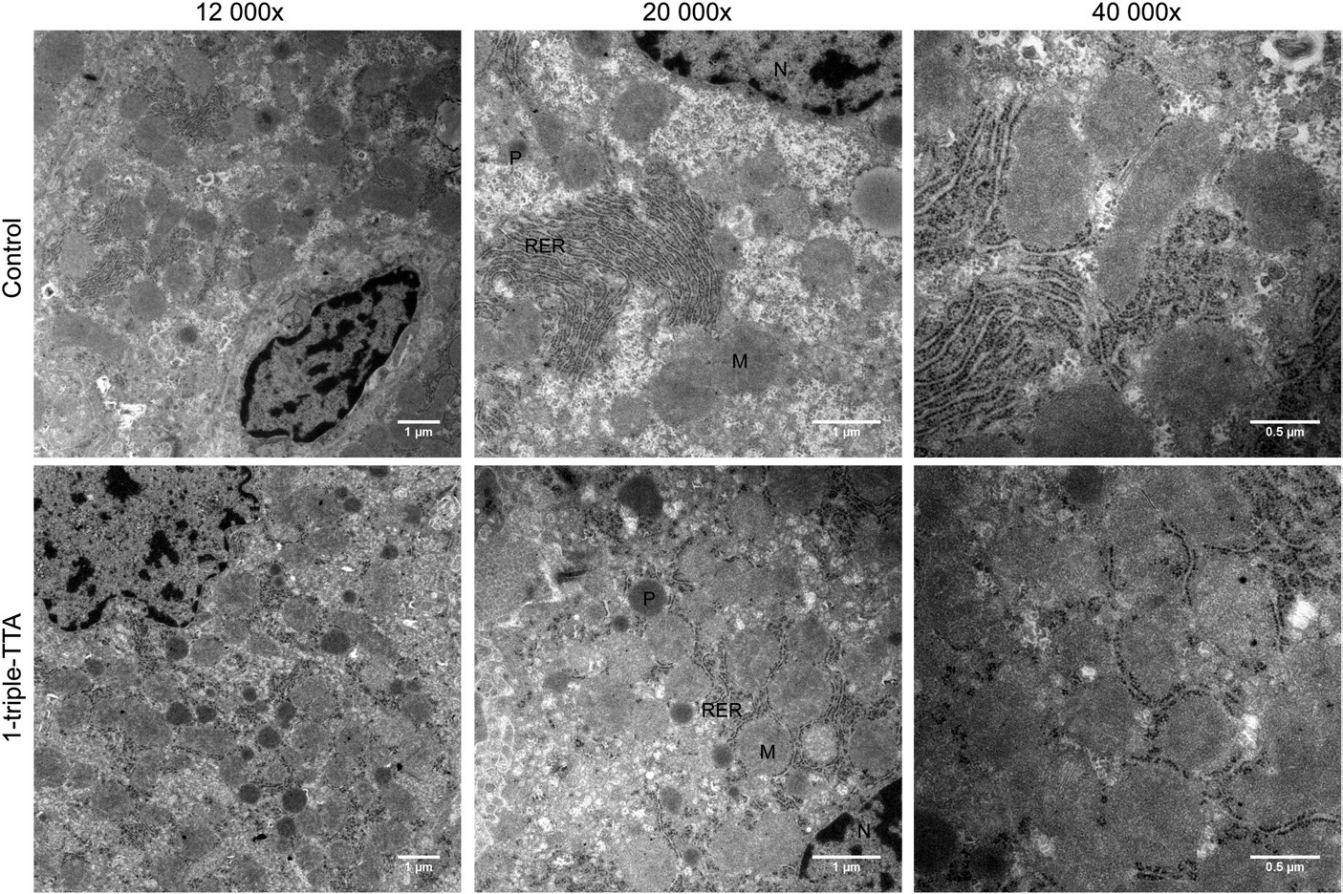
TEM of liver from male Wistar rats. Representative samples are shown from control (upper row) and 1-triple TTA-treated rats (100 mg 1-triple TTA/kg body weight for 21 days; second row). The magnification used for image acquisition is indicated (12,000×, 20,000×, and 40,000×). M, mitochondria; N, nucleus; P, peroxisomes.

**Fig. 8.**
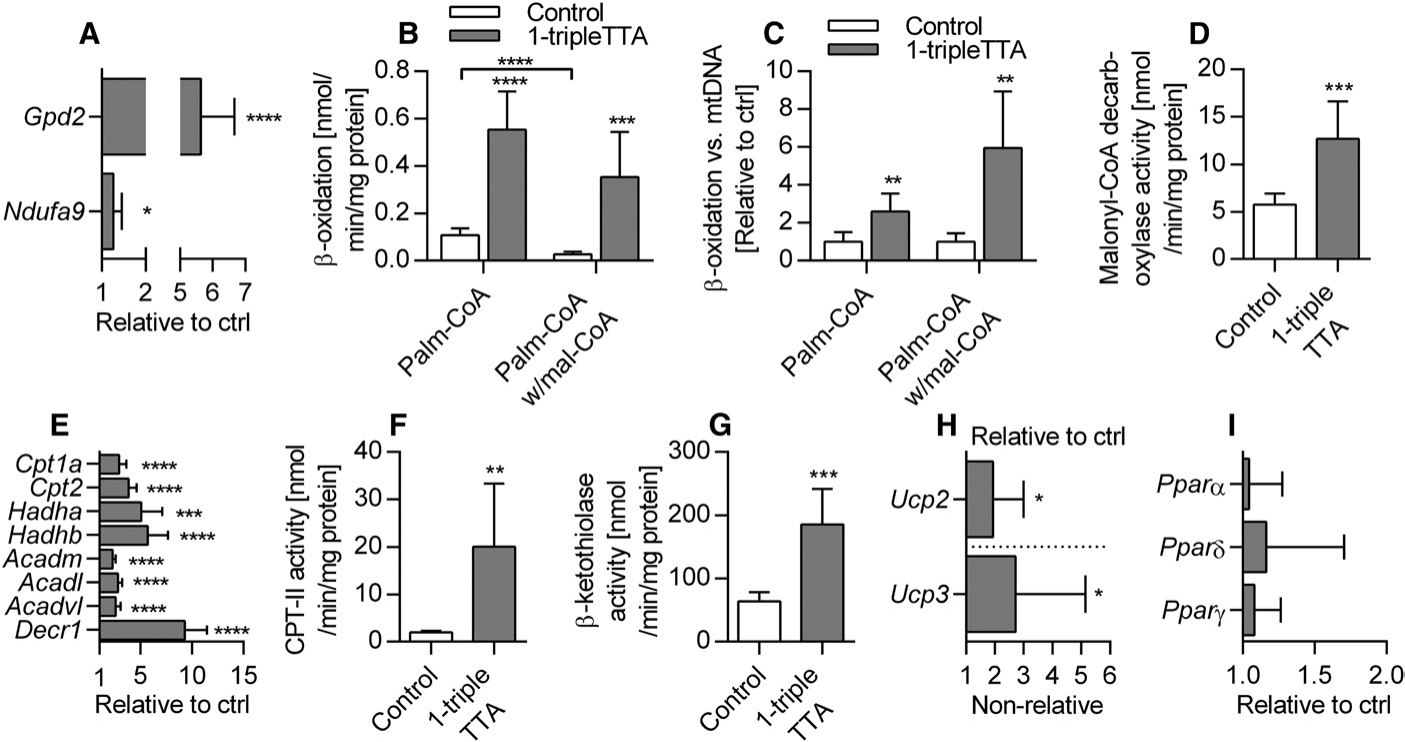
Mitochondrial activity and biomarkers in Wistar male rats given 100 mg/kg 1-triple TTA for 3 weeks compared with controls. A: Hepatic mRNA levels of mitochondrial biomarkers, mitochondrial *Gpd2* and NADH dehydrogenase (ubiquinone) 1α subcomplex 9 (*Ndufa9*), shown relative to control. B: Hepatic palmitoyl-CoA oxidation with or without malonyl-CoA. C: Hepatic palmitoyl-CoA oxidation with or without malonyl-CoA, related to mtDNA, set as relative to control. D: Hepatic malonyl-CoA decarboxylase activity. E: Hepatic mRNA levels of enzymes involved in β-oxidation: *Cp1a* and *Cpt2*; hydroxyacyl-CoA dehydrogenase/β-ketothiolase/enoyl-CoA hydratase (trifunctional protein) α and β subunit (*Hadha*, *Hadhb*); *Acadm*, *Acadl*, *Acadvl*; and mitochondrial *Decr1* shown relative to the control. F: Hepatic CPT-II activity. G: Hepatic β-ketothiolase activity. H: Hepatic mRNA levels of *Ucp2* and *Ucp3*. *Ucp2* is relative to control. *Ucp3* is only normalized to 18S. I: Hepatic mRNA levels of PPARα, PPARδ, and PPARγ shown as relative to the control. Values are shown as mean ± SD (n = 5–8). Significant difference was determined with Student’s *t*-test (*P ≤ 0.05, **P ≤ 0.01, ***P ≤ 0.001, ****P ≤ 0.0001).

Because 1-triple TTA administration affected energy parameters in a tissue-specific manner, we next evaluated the effects on liver mitochondrial function and biogenesis. The liver index was increased by 1-triple TTA (**Fig. 6A**) and a significantly higher level of mtDNA relative to nDNA was found in the livers of 1-triple TTA fed rats, but not in heart and skeletal muscle (Fig. 6B). The weights of heart (Fig. 6C) and epididymal white adipose tissue (Fig. 6D) were unchanged by 1-triple TTA administration. Induction of mitochondrial biogenesis in the livers of 1-triple TTA-treated rats was further supported by the increase of the mitochondrial biomarkers citrate synthase activity (Fig. 6E), cytochrome c (*Cycs*) gene expression, and TFAM gene expression, although *Ppargc1a* mRNA was not increased (Fig. 6F). Citrate synthase activity was unchanged when related to mtDNA (data not shown), demonstrating that its increase (Fig. 6E) was linked to mitochondrial proliferation. Transmission electron microscopy (TEM) was performed on liver tissue samples from control and 1-triple-TTA-treated rats and subcellular morphology was compared. Although the samples were prepared from snap-frozen biopsies, the structures were generally intact and differences could be observed between controls and 1-triple TTA-treated rats (**Fig. 7**). In control samples, rough endoplasmic reticulum (RER) had the classical appearance, with ribosomes attached to a folded ER membrane, often forming a stack at specific localizations in the cytoplasm. In the 1-triple TTA-treated rats, the RER structures did not tend to form stacks and, therefore, had a more delocalized appearance, often between or around mitochondrial organelles. Mitochondria with classic spherical morphology predominated in both groups. In these samples, the number of both mitochondria and peroxisomes appeared to be somewhat increased in the 1-triple-TTA-treated group compared with control. To explore the implications of these findings, liver energy metabolism was investigated in more detail. The mRNA level of mitochondrial *Gpd2* and a subunit of NADH dehydrogenase, *Ndufa9*, was increased by 1-triple TTA administration (**Fig. 8A**). The findings, so far, suggested that 1-triple TTA mediates a general induction of enzymes involved in mitochondrial energy production. As mitochondrial FA oxidation serves to fuel oxidative phosphorylation, we next investigated to determine whether 1-triple TTA caused increased oxidation of palmitoyl-CoA in liver. Confirming this hypothesis, 1-triple TTA was found to increase hepatic FA oxidation, calculated in relation to protein (Fig. 8B) and mtDNA (Fig. 8C). The 1-triple TTA was also found to reduce the inhibitory effect of malonyl-CoA on FA oxidation (Fig. 8B, C). Increased β-oxidation was accompanied by an increased activity of malonyl-CoA decarboxylase (Fig. 8D). In line with these data, we observed, in the livers of rats treated with 1-triple TTA, a significant increase in mRNA level of *Cpt1a* and *Cpt2*, α and β subunits of the mitochondrial trifunctional protein (*Hadha* and *Hadhb*), *Acadm*, *Acadl*, *Acadvl*, and *Decr1*, an enzyme involved in β-oxidation of polyunsaturated FAs (Fig. 8E). This was associated with increased activity of CPT-II (Fig. 8F) and β-ketothiolase of the trifunctional protein (Fig. 8G), as well as higher gene expression of UCP2 and UCP3 (Fig. 8H). Several of these genes are known to be regulated by the PPAR transcription factor family, suggesting that 1-triple TTA causes PPAR activation. The gene expression of the three isoforms of PPAR remained constant after 1-triple TTA administration (Fig. 8I).

### 1-triple TTA lowers TG accumulation in liver

**Fig. 9.**
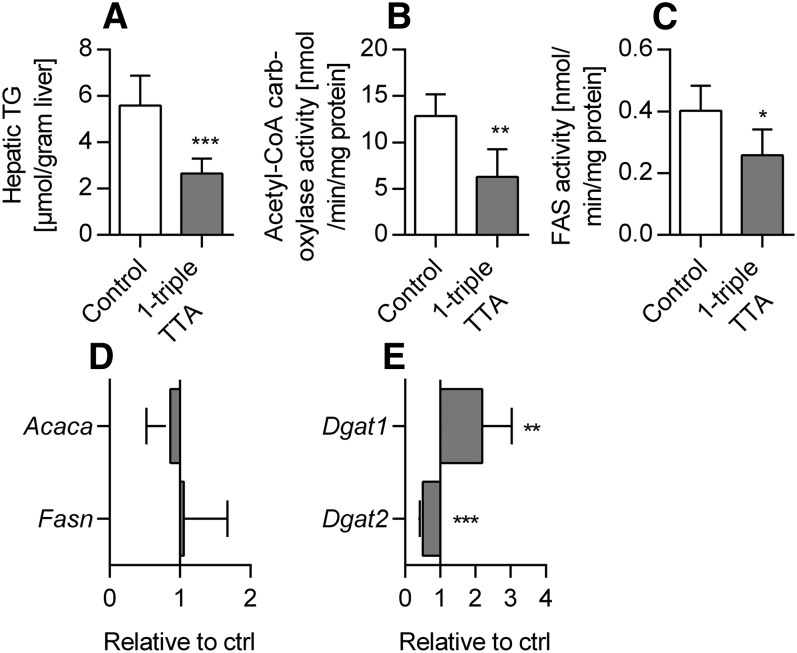
Hepatic lipid levels, enzyme activities, and mRNA levels related to lipogenesis and plasma insulin in Wistar male rats given 100 mg/kg 1-triple TTA for three weeks compared with controls. A: Hepatic TG content. B: Hepatic acetyl-CoA carboxylase activity. C: Hepatic FAS activity. D: Hepatic mRNA levels of acetyl-CoA carboxylase (*Acaca*) and *Fasn*, shown as relative to control. E: Hepatic mRNA levels of *Dgat1* and* Dgat2* shown as relative to control. Values are shown as mean ± SD (n = 5–8). Significant difference was determined with Student’s *t*-test (*P ≤ 0.05, **P ≤ 0.01, ***P ≤ 0.001, ****P ≤ 0.0001).

Given the relevance of mitochondrial FA oxidation in regulation of both liver and plasma TG, we next wondered how 1-triple TTA affected TG metabolism in rat liver. The hepatic TG level was reduced by 53% and was accompanied by a reduction in the activities of acetyl-CoA carboxylase and FAS (**Fig. 9A–C**). However, the gene expressions (*Acaca* and *Fasn*) were not changed (Fig. 9D). It is noteworthy that a negative correlation of liver TG and palmitoyl-CoA oxidation, as well as a positive correlation between liver TG and acetyl-CoA carboxylase activity, was observed (**Table 1**). Moreover, the mRNA level of *Dgat1* was increased by 1-triple TTA, whereas *Dgat2* was decreased (Fig. 9E).

**Table 1.** Pearson correlation coefficients

Parameters	Palmitoyl-CoA Oxidation	FAS Activity	Acetyl-CoA Carboxylase Activity	mRNA *ApoC-III*
Plasma TG	−0.7456*^a^*	0.8263*^b^*	0.6547*^c^*	0.6499*^c^*
Hepatic TG	−0.7921*^a^*		0.6742*^c^*	
mRNA *ApoC-III*	−0.8873*^d^*			

Pearson correlation coefficients of mitochondrial β-oxidation and TG in liver, mitochondrial β-oxidation and TG in plasma, FAS activity and TG in liver, hepatic FAS activity and TG in plasma and mitochondrial β-oxidation and hepatic mRNA *ApoC-III* in Wistar male rats fed 100 mg/kg 1-triple TTA for 3 weeks. Values are shown as correlation coefficients (r), (n = 5–8). Significance of coefficients are shown.

a *P* ≤ 0.01.

b *P* ≤ 0.001.

c *P* ≤ 0.05.

d *P* ≤ 0.0001.

### 1-triple TTA lowers plasma TG concentration

**Fig. 10.**
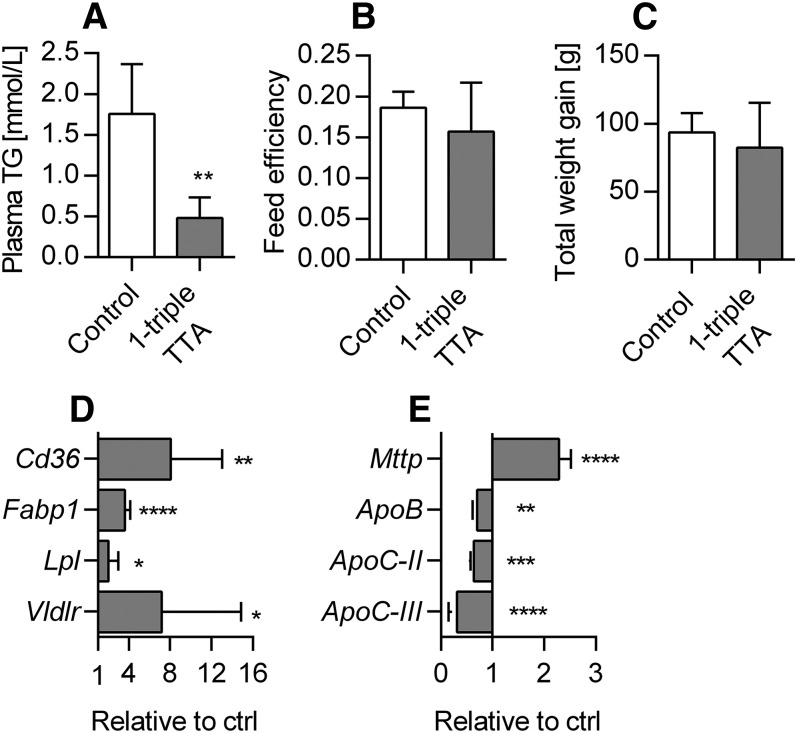
Plasma levels and enzyme activities in muscle and heart tissue in Wistar male rats given 100 mg/kg 1-triple TTA for 3 weeks compared with controls. A: Plasma TG concentration. B: Feed conversion efficiency [change in body weight (g)/feed intake (g)]. C: Total body weight gain. D: Hepatic mRNA levels of the fatty acid translocase, *Cd36*, *Fabp1*, *Vldlr*, and *Lpl* shown as relative to control. E: Hepatic mRNA levels of *Mttp*, *ApoB*, *ApoC-II*, and* ApoC-III*. Values are shown as means and SD (n = 5–8). Significant difference compared with controls was determined with Student’s *t*-test (*P ≤ 0.05, **P ≤ 0.01, ***P ≤ 0.001, ****P ≤ 0.0001).

The plasma TG level was reduced by more than 70% in 1-triple TTA-treated rats compared with controls (**Fig. 10A**), which was not caused by changes in feed intake (data not shown) or feed efficiency, as this remained constant (Fig. 10B). No significant reduction in body weight was observed during the 3 week study (Fig. 10C). Because the blood TG level is determined by a delicate balance between hepatic TG synthesis and secretion, as well as plasma clearance, we were prompted to test whether 1-triple TTA could affect hepatic genes related to FA uptake and secretion. The mRNA levels of *Cd36* and *Fabp1* were highly significantly increased (Fig. 10D), and the mRNA levels of *ApoB* and *Mttp* (i.e., subunit of MTP) were decreased and increased, respectively, by 1-triple TTA administration compared with controls (Fig. 10E). The gene expression of hepatic LPL was also significantly increased (Fig. 10D), whereas the mRNA levels of *ApoC-II* and, especially, *ApoC-III* were decreased compared with controls (Fig. 10E). This was associated with an increased mRNA level of *Vldlr* (Fig. 10D). It is noteworthy that this resulted in a negative correlation between mitochondrial FA oxidation and the *ApoC-III* mRNA level and a positive correlation between *ApoC-III* and plasma TG (Table 1). Moreover, the plasma TG concentration correlated negatively with palmitoyl-CoA oxidation and positively with acetyl-CoA carboxylase and FAS activity (Table 1). Thus the 1-triple TTA-mediated clearance of blood TG seems to partly be a result of lowered APOC-III with a subsequent induction of LPL and (re)uptake of FAs from VLDL. Whether 1-triple TTA also decreased hepatic VLDL secretion should be considered because APOB gene expression was decreased.

### 1-triple TTA increases plasma ketone bodies

**Fig. 11.**
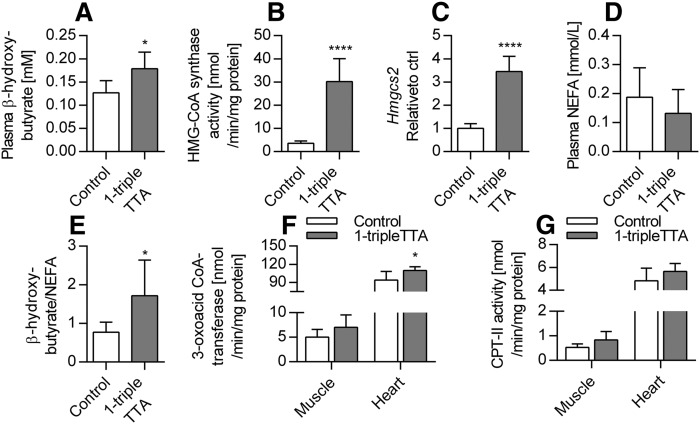
Hepatic ketone body production and muscle and cardiac enzyme activities in Wistar male rats given 100 mg/kg 1-triple TTA for 3 weeks compared with controls. A: Mitochondrial HMG-CoA synthase activity in liver. B: Hepatic mRNA level of mitochondrial *Hmgcs2*. C: NEFA plasma concentration. D: Plasma concentration of the ketone body, β-hydroxybutyrate. E: Ratio of β-hydroxybutyrate and NEFA. F: Oxalate CoA-transferase activity in muscle and heart tissue. G: CPT-II activity in muscle and heart tissue. Values are shown as means and SD (n = 5–8). Significant difference compared with controls was determined with Student’s *t*-test (*P ≤ 0.05, ****P ≤ 0.0001).

The liver is able to produce ketone bodies from acetyl-CoA, which may be used as an alternative fuel by other tissues. It is of interest that 1-triple TTA significantly increased the plasma level of β-hydroxybutyrate accompanied by increased hepatic activity and mRNA level of the mitochondrial *Hmgcs2* (**Fig. 11A–C**). Accordingly, because the concentration of NEFAs was unchanged (Fig. 10D), a significant increase in the β-hydroxybutyrate/NEFA ratio was found in 1-triple TTA-treated rats compared with controls (Fig. 11E). The ketone bodies were taken up by extrahepatic tissue, such as skeletal and cardiac muscle, where they were converted to CoA derivatives for oxidative metabolism. It is noteworthy that the activity of oxalate-CoA transferase, an enzyme involved in the oxidation process of β-hydroxybutyrate, was significantly increased in heart, but not muscle, while CPT-II activity was marginally increased in muscle, but not heart (Fig. 11F, G). Hence, in the livers of 1-triple TTA-treated animals, the increased mitochondrial FA oxidation appears to fuel the formation of ketone bodies, which may be an efficient source of energy for other tissues.

## DISCUSSION

**Fig. 12.**
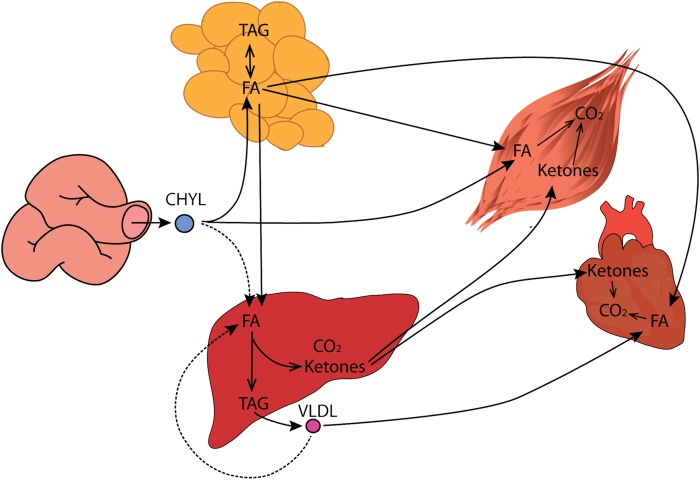
Scheme of the drainage hypothesis provoked by 1-triple TTA. Due to increased mitochondrial function, TG is drained from the blood toward the liver for catabolism leading to production of ketone bodies for extrahepatic fuel. The dashed arrows demonstrate a theory that 1-triple TTA may induce uptake of TG from the intestine as well. TAG, triacylglycerol.

This work demonstrates that the mitochondrial targeted compound, 1-triple TTA, leads to enhanced hepatic FA oxidation and has the potential to protect against TG accumulation in liver and plasma. The observed increase in mitochondrial FA oxidation capacity and ketogenesis in liver can be explained by induction of mitochondrial biogenesis accompanied by increased oxidative phosphorylation and/or metabolism. These findings are associated with altered energy state parameters in a tissue-specific manner and possibly with moderate uncoupling, as hepatic mRNA levels of *Ucp2* and *Ucp3* were increased. The decreased hepatic TG content in 1-triple TTA-treated rats was most likely related to increased FA oxidation, decreased lipogenesis, and reduced TG synthesis. The plasma TG was also reduced by 1-triple TTA treatment and this was accompanied by an increased level of ketone bodies in plasma. The 1-triple TTA-mediated clearance of blood TG may result from lowered APOC-III levels, subsequent increased hepatic LPL, mitochondrial FA oxidation, and (re)uptake of FAs from VLDL, thereby draining FAs from blood to the liver for β-oxidation and production of ketone bodies for extrahepatic fuel (**Fig. 12**). Under these conditions, we also hypothesize that TG-rich lipoproteins, including CHYLs, will be drained from intestine to liver.

CHYLs formed in the enterocytes of the small intestine are transported into the main circulation through the ductus thoracicus and bypass the portal circulation and liver. The TG-enriched CHYLs are hydrolyzed in adipose tissue and in muscle by LPL attached to the capillary walls. Therefore, TG from CHYLs is normally not taken up in the liver to a great extent, as LPL is not expressed in this tissue (43). However, some of the NEFAs formed by LPL enter the circulation and reach the liver through the hepatic artery. It is known that regulation of MTP is rather complex (44), but it is usually correlated with plasma TG levels (45). Although further studies are needed to elucidate the influence of 1-triple TTA on lipoprotein assembly and secretion, the data presented suggest that 1-triple TTA does not lower TG levels by inhibition at the transcriptional level. Further, 1-triple TTA decreased the hepatic mRNA level of *ApoC-III*, but increased the hepatic gene expression of LPL and the VLDL receptor. Hence, a hypothesis is presented in Fig. 12 that both CHYLs and VLDLs may directly be taken up by the liver and, thus, establish a direct route of FA transport from the intestine to the liver and/or reabsorption of VLDL formed in the liver itself (Fig. 12). However, further studies are needed to elucidate this hypothesis. The uptake of NEFA in the liver is relatively small in relation to total NEFA turnover. Therefore, the liver has little immediate influence on blood NEFA levels; but after 1-triple TTA treatment, stimulated FA oxidation and ketogenesis in the liver will drain FAs from blood.

It is known that PPARα is more prominent in liver; hence, it indicates that the liver-specific function of 1-triple TTA is due to PPARα activation. Further, increased in vitro palmitoyl-CoA oxidation along with increased enzyme activities and mRNA levels of enzymes involved in mitochondrial β-oxidation (Fig. 8) are well-known PPAR responsive effects/genes (46). The gene expression of PPAR isoforms was not altered by 1-triple TTA. Nevertheless, 1-triple TTA increased mitochondrial FA β-oxidation and liver size, a well-known effect of PPARα activation in rodents (47). PPAR-induced FA oxidation has previously been associated with increased L-carnitine and acetylcarnitine in plasma (47, 48), and with increased mRNA levels of *Bbox1* and *Slc22a5* (47, 49); while, in this study, hepatic *Bbox1* mRNA and plasma L-carnitine and acetylcarnitine were decreased. In contrast, mRNA levels of *Crat* and the carnitine transporter, *Slc22a5*, were increased as a consequence of 1-triple TTA treatment (Fig. 2). These results imply an efficient carnitine turnover sufficient for the observed increase in palmitoyl-CoA oxidation, partly unrelated to PPAR activation. The increase in mitochondrial β-oxidation and the decrease in plasma acetylcarnitine were accompanied by increased HMG-CoA synthase activity and plasma level of ketone bodies. This indicates efficient removal of the final β-oxidation product, acetyl-CoA, by its conversion to ketone bodies. Extrahepatic tissues from 1-triple TTA-treated rats may preferably use ketone bodies as fuel, because the activity of the enzyme involved in ketone body utilization, oxalate CoA-transferase, was increased in 1-triple TTA-treated rats, while CPT-II activity was decreased in heart and vice versa in muscle.

Plasma TG was significantly reduced in 1-triple TTA-treated rats accompanied by reduced hepatic mRNA levels of *ApoB*, suggesting reduction of VLDL secretion. We found a positive correlation between hepatic *ApoC-III* mRNA and plasma TG, a negative correlation between *ApoC-III* mRNA and hepatic palmitoyl-CoA oxidation, and a negative correlation between palmitoyl-CoA oxidation and plasma TG levels (Table 1). This is in agreement with previous findings that increased hepatic mitochondrial FA oxidation is involved in the regulation of plasma TG (50). In addition, it is hypothesized that plasma APOC-III could be negatively associated with stimulated mitochondrial FA oxidation. The hepatic TG concentration was also reduced by 1-triple-TTA, accompanied by increased mRNA levels of *Cd36* and *Fabp1*, decreased activities of acetyl-CoA carboxylase and FAS, and reduced gene expression of DGAT2. Assuming that FA flux pathways are associated with changes in mRNA levels, FAs are efficiently oxidized and chain-shortened in mitochondria due to an increased flux (see above) to the liver resulting in a decrease of hepatic TG. This observed decrease in TG may also be partly due to decreased lipogenesis and subsequently reduced TG biosynthesis, as it is evident that DGAT2 is responsible for the majority of TG synthesis (51). Both DGAT1 and DGAT2 reside in the ER, in which DGAT2 also colocalizes with mitochondria (52) and lipid droplets (53). Whether 1-triple TTA reduces mitochondria-associated TG biosynthesis should be considered.

Mitochondrial function and biogenesis depend on regulatory coordination between nuclear and mitochondrial genomes (54, 55). TFAM is an essential factor for mtDNA replication and expression (56). The increase of hepatic *Tfam*, *Cycs*, *Ndufa9* (Figs. 6, 8), *Atp5c1*, and *Atp5j2* (Fig. 3) mRNA levels accompanied by increased mtDNA and citrate synthase activity (Fig. 6F), as well as more mitochondria observed by TEM (Fig. 7), indicate that 1-triple TTA administration induces mitochondrial biogenesis even though gene expression of PGC-1α (*Ppargc1a*) was not affected (Fig. 6F). Examination of morphology studies also showed changes in subcellular organization, which have been found to be associated with induction of a hypertrophic response involving mitochondrial biogenesis (40). Although some aspects of the intracellular morphology appeared less organized in samples from 1-triple TTA-treated rats, possibly due to hypertrophy, there were no signs of cellular toxicity. β-Oxidation was increased by 1-triple TTA compared with control, both in relation to milligrams of protein and in relation to mtDNA. This implies that, although 1-triple TTA leads to mitochondrial proliferation, the inducement of mitochondrial activity is partly independent of this proliferation. Induction of mitochondrial biogenesis may increase the capacities of mitochondrial respiration and FA oxidation to support ATP production. Reducing equivalents (electrons) from FA oxidation enter the respiratory chain at the level of complex I and coenzyme Q. Mitochondrial glycerol-3-phosphate dehydrogenase is a peripheral component of the glycerophosphate shuttle, in which electrons from cytosolic NADH can enter the mitochondrial respiratory chain at the level of coenzyme Q. This enzyme, which was strongly upregulated in the liver after 1-triple TTA administration (Fig. 8), may function as an important link between mitochondrial and cytosolic energy pathways and also influence the cellular redox state (57). Increased gene expression of UCP2 and UCP3 suggested that respiratory uncoupling contributes to decreased ATP yield, and may therefore explain the decreased energy charge. Similar observations were made previously after treatment with the modified FA, TTA (19), where proton electrochemical potential was lowered without alteration of ΔpH. Moreover, an increase in AMP is known to activate AMPK, which is an energy sensor, serving to support ATP production under conditions of energy depletion (58). The hepatic mRNA levels of two genes encoding the catalytic subunit of AMPK were either unchanged or decreased (Fig. 3) and no change in the AMPK phosphorylation level was found with 1-triple-TTA treatment. The degradation product of adenine, hypoxanthine, was increased, whereas xanthine was decreased compared with controls. This indicates that there is a partial inhibition of xanthinoxidase with 1-triple-TTA treatment in the liver and, as a consequence, reduced superoxide production by the enzyme.

In conclusion, increased hepatic mitochondrial FA oxidation was found to explain the TG-lowering effects observed in 1-triple TTA-treated rats. This mechanism was further supported by upregulation of mitochondrial biogenesis and the oxidative machinery, and remodeling of the lipoprotein profile. Further, the data indicate that 1-triple TTA causes a pseudo-fasted state in rat liver, with a reduced yield of ATP caused by what we assume to be a moderate degree of respiratory uncoupling. Further studies are required to evaluate the potential use of 1-triple TTA to target pathogenic mechanisms in disorders such as the metabolic syndrome, nonalcoholic fatty liver disease, and CVD.
